# Towards Ultra-Precision Manufacturing: Advancements and Future Trends in Energy Field-Assisted Jet Machining

**DOI:** 10.3390/mi17040415

**Published:** 2026-03-29

**Authors:** Yongzhen He, Ting’an Chen, Xinhua Man, Tonglu Su

**Affiliations:** School of Mechanical and Electronic Engineering, Shandong Jianzhu University, Jinan 250101, China

**Keywords:** waterjet machining, multi-energy field, laser assistance, ultrasonic assistance, magnetic assistance, precision manufacturing

## Abstract

Jet machining is widely utilized in innovative technology industries, such as aerospace and semiconductors, due to its minimal thermal damage. However, with the increasingly stringent surface quality requirements of modern manufacturing, conventional jet technologies face limitations in achieving ultra-precision surface finishing and high material removal rates. To address these challenges and adapt to this new situation, multi-energy field-assisted jet machining has emerged as a novel concept, integrating laser, ultrasonic, and magnetic fields. This paper reviews the scientific development and recent advancements of these hybrid technologies within the field of ultra-precision machining. The physical interaction mechanisms between the auxiliary energy fields and the waterjet are elucidated. Specifically, the effects of laser thermal softening, ultrasonic cavitation, and magnetic focusing on new mechanisms of material removal and surface topography are systematically analyzed. The process capabilities and applications of each method are evaluated. Finally, current technical challenges are identified, and the future trends in ultra-precision jet machining are discussed.

## 1. Introduction

**Figure 1:**
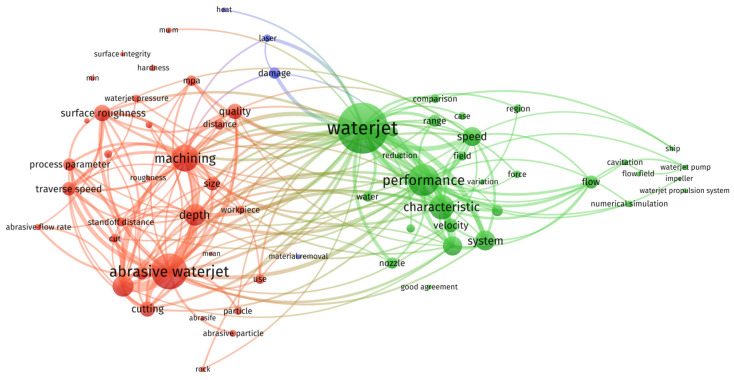
Waterjet Machining Research Data Correlation Diagram.

With the advancement of high-end equipment manufacturing, the demand for micro-devices and functional surface microstructures has increased. Components such as 316L stainless steel micro-traps, K9 glass micro-holes, Ti-6Al-4V alloy Products [1] and Atmospheric Plasma Spray (APS) coatings are utilized in particle accelerators, aerospace, and information electronics. These components are characterized by high integration, miniature dimensions, and superior material properties. However, these components present significant machining challenges due to their structural complexity, strict tolerance requirements, and poor machinability. As a non-contact manufacturing method, jet machining is applied to prevent thermal damage and ensure material compatibility [2,3]. The data correlation map within the waterjet machining research field is illustrated in Figure 1. Through this analysis, major research themes and their interdependencies are identified, and key areas such as process optimization, machining simulation, and parameter control are emphasized [4].

Waterjet technology is utilized as a tool for micro-machining [5]. High-velocity liquid droplets or abrasive particles impact the workpiece surface to erode the material [6,7]. Excess material is removed from the target area via this erosion mechanism [8,9]. Compared to conventional techniques, advantages such as the absence of thermal deformation, high flexibility, and broad material applicability are offered. Furthermore, integration with mechanical systems is facilitated, and a balance between surface roughness and residual stress is maintained [10]. Low cutting forces with minimal burr formation are ensured [11]. Both pure waterjet and abrasive waterjet technologies are investigated in manufacturing research [12,13,14,15]. However, restricted to a single mechanical energy form, conventional waterjet machining struggles to meet the demands of ultra-precision manufacturing, facing inherent trade-offs between a high material removal rate and ultra-smooth surface integrity, and there are obvious shortcomings in the processing of hard-brittle materials, high-aspect-ratio microstructures and complex curved components. To break through these bottlenecks, multi-energy field-assisted jet machining, which couples laser, ultrasonic and magnetic fields with traditional waterjet technology, has gradually become a research hotspot in the field of ultra-precision manufacturing.

**Figure 2:**
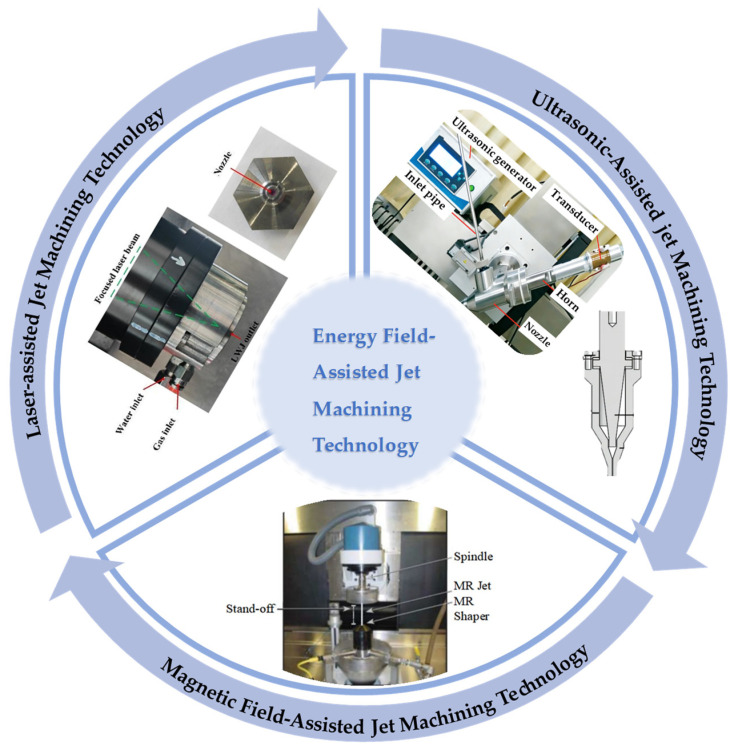
Schematic diagram of energy field-assisted machining [17,22,24].

Consequently, the multi-energy field-assisted jet machining technology shown in Figure 2 has been developed, and the process is optimized through synergistic effects. In laser-assisted waterjet technology, the material is preheated by a laser beam [16,17]. The surface is softened by thermal energy, reducing shear strength. Subsequently, material is removed by the mechanical action of the high-speed waterjet. This method is applied to hard, brittle, or high-strength materials [18,19,20]. The required impact energy is reduced by laser heating. Simultaneously, softened material is flushed away by the waterjet, preventing residual thermal damage. This technique is suitable for the fabrication of fine structures.

Ultrasonic-assisted abrasive waterjet technology utilizes ultrasonic vibration to induce periodic pulsations in the jet [21]. Ultrasonic vibration transforms the continuous jet into a pulsed jet, significantly enhancing dynamic pressure and cavitation effects. The kinetic energy of abrasive particles is increased by the transmission of vibration. Furthermore, high-pressure micro-jets are generated by the collapse of cavitation bubbles, and micro-erosion on the workpiece surface is intensified [22]. Processing efficiency for hard-brittle materials, such as glass and ceramics, is improved. Additionally, surface flatness is enhanced, and morphological defects are mitigated [23].

Magnetic forces constrain the trajectory of magnetic abrasives, thereby reducing particle divergence [24]. Consequently, focusing performance and energy concentration are improved. Abrasives are aggregated near the jet axis, enhancing the impact effect on the target zone. Damage to non-machining areas is minimized, and the technology is suitable for the formation of high-precision micro-structures.

Currently, advantages are demonstrated by single-energy techniques for specific materials [25,26]. However, technical challenges regarding multi-energy field coupling mechanisms, parameter synergy, and equipment integration remain. Large-scale manufacturing demands are not yet fully met, and further research is required.

Existing review articles have primarily focused on single-energy field assistance. For instance, laser-assisted waterjet technologies are frequently discussed in isolation [27]. However, a comprehensive comparative analysis of multiple energy fields is currently lacking in the literature [28]. To address this gap, a systematic review is presented in this paper. The synergistic reinforcement mechanisms of laser, ultrasonic, and magnetic fields are individually analyzed and compared. Furthermore, the specific process capabilities and limitations of each method are evaluated. A holistic perspective on multi-energy field coupling is thus provided for high-performance manufacturing. The structure of this paper is organized as follows. First, the requirements for energy field-assisted machining are addressed. Core processing technical challenges and coupling mechanisms are analyzed. Second, three types of energy field-assisted micro-machining—acoustic, magnetic, and laser—are reviewed. Process principles and applications are summarized. Finally, development trends are synthesized, and future directions are discussed.

## 2. Challenges and Mechanisms of Multi-Energy Field-Assisted Jet Machining Technology

### 2.1. Technical Challenges in High-Performance Manufacturing with Jet Machining

#### 2.1.1. Limitations in Material Performance and Machinability

High-performance materials, represented by semiconductor crystals, optical glass, and superalloys, possess superior service characteristics. Advantages such as ultra-high hardness, low fracture toughness, and resistance to high temperatures are exhibited in engineering applications [29]. However, vulnerability during processing is induced by their specific microstructural properties. When a high-kinetic-energy, high-pressure waterjet (HPWJ) impacts the material surface, it disrupts atomic bonding structures at the microscopic level. Chain-reaction brittle fracture is triggered [30]. Edge chipping defects are generated, which not only destroy geometric integrity but also act as sources of stress concentration.

Regarding superalloys, during HPWJ machining, the continuous impact of abrasive particles forms a work-hardened layer on the material surface. The chemical state is simultaneously altered. While residual compressive stress is generated in the surface layer, surface roughening or micro-crack defects are introduced if process parameters are improperly controlled [31].

The mechanism of this limitation lies in the energy transfer of the water stream. The critical point from brittle fracture to plastic deformation cannot be breached by hydraulic energy alone. More microscopic, controllable removal is difficult to achieve [32]. If dislocation slip is not activated by sufficient jet energy, material removal is realized solely through fracture [33]. This constitutes a fundamental physical limit for waterjets in the field of ultra-precision machining.

#### 2.1.2. Limitations in Workpiece Microstructure and Machining Precision

A significant contradiction exists between the physical characteristics of waterjets and the requirements for micro-scale precision. This is particularly evident in the fabrication of precision micro-channels, optical micro-structures, or bio-inspired functional surfaces. The jet exhibits an umbrella-shaped diffusion effect after exiting the nozzle [34]. Under the dual effects of energy attenuation and beam scattering, vertical sidewalls are distorted into inclined features. The machining profile is difficult to predict effectively. Consequently, erosion models are challenging to establish.

For precision micro-lenses, the ideal curvature is deformed under non-uniform erosion. Optical focusing performance is significantly degraded. Distortion appears in the micro-topological features of bio-inspired structures. Functional wettability properties deviate from design targets; for instance, hydrophilic surfaces may transition to a hydrophobic state. The root of this precision loss lies in the scale mismatch between the tool and the workpiece. The waterjet behaves as a macro-scale flexible energy carrier, following the laws of continuum mechanics. However, functional micro-structures require rigid energy focusing and precise action at the microscopic scale.

#### 2.1.3. Limitations in Workpiece Configuration and Tool Adaptability

The limitations of traditional waterjet tool systems are highlighted by the processing of emerging complex structures. When the water beam penetrates narrow cavities, secondary collisions occur. Kinetic energy decays exponentially with depth [35]. A chaotic flow field is formed by multiple reflections between cavity walls. Consequently, an unpredictable gradient distribution is presented by the material removal rate [36]. Bottom profiles are deformed due to insufficient energy, and sharp edges are transformed into arc transitions [37].

When processing complex surfaces with significant curvature changes, the nozzle stand-off distance fluctuates dynamically. This fluctuation destabilizes the jet impact and causes uneven material removal, making it difficult to maintain consistent surface quality.

#### 2.1.4. Limitations in Surface Integrity and Machining Efficiency

As machining precision requirements become increasingly stringent, demands on waterjet technology are intensified. Jet pressure directly dictates flow velocity, which in turn influences the kinetic energy of entrained particles [38]. Consequently, an increase in pressure leads to higher material removal rates (MRR) but also increases surface roughness [39]. Surface quality can be improved by reducing the feed rate; however, production efficiency is significantly compromised. Extended machining times trigger other systemic issues. Micro-abrasives are more prone to accumulation and clogging under low-speed conditions or in the presence of an air phase. Additionally, nozzle apertures are progressively enlarged due to continuous abrasion, resulting in batch-to-batch quality fluctuations.

**Figure 3:**
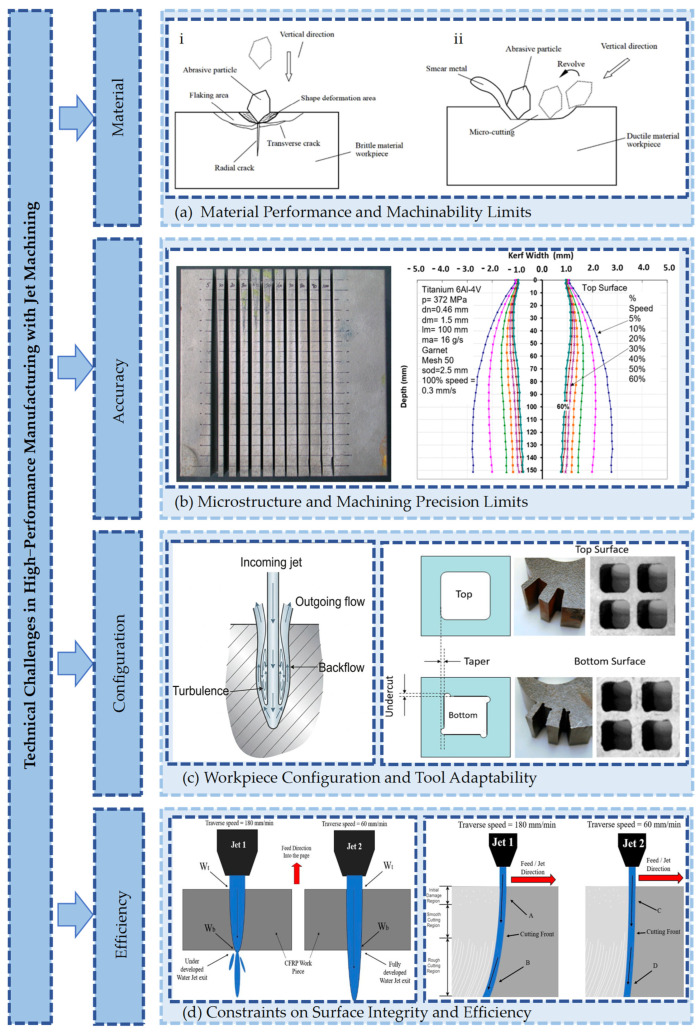
Schematic of Technical Challenges in High-Performance Manufacturing via Jet Machining [32,34,39].

In summary, the core limitations of waterjet machining technology stem from the inherent conflict between the macro-scale continuous medium energy carrier and the requirements of micro-precision machining (as shown in Figure 3). The essence of these limitations is the physical restriction of a single hydraulic energy form in terms of control accuracy and action mechanism [40].

### 2.2. Synergistic Reinforcement Mechanisms of Mechanical and Specialized Energy Fields

#### 2.2.1. Laser-Mechanical Synergistic Reinforcement

**Figure 4:**
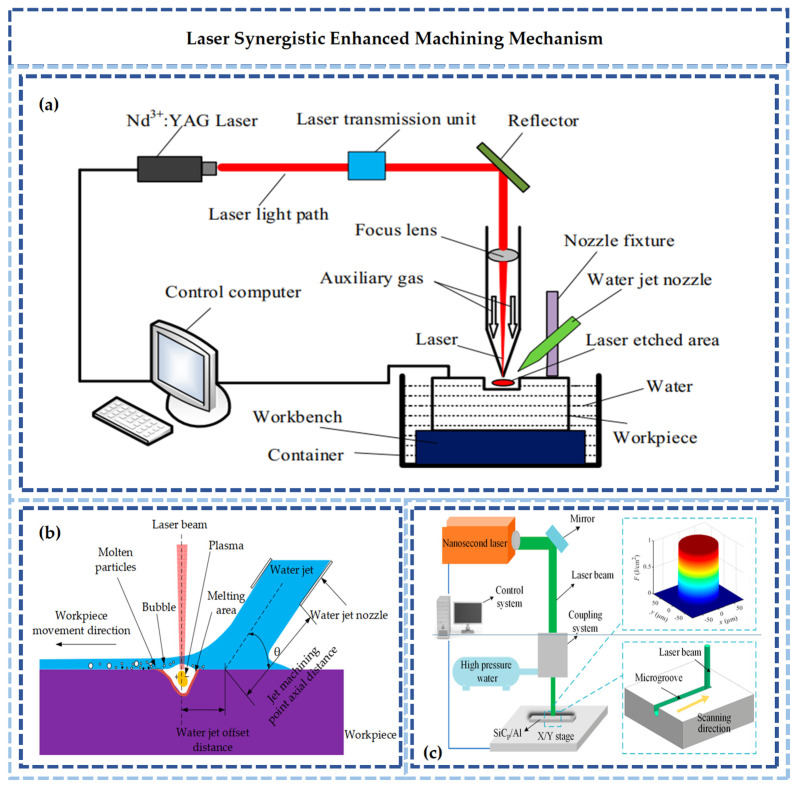
Schematic diagram of laser–mechanical energy synergistic strengthening: (**a**) Laser–Water Jet Synergistic Machining Setup [43]. (**b**) Laser–Water Jet Synergistic Material Removal Mechanism [43]. (**c**) Nanosecond Laser Microgroove Machining [44].

The synergistic mechanism between laser and mechanical energy is characterized as a sequential process of “photothermal pretreatment followed by hydraulic precision removal.” As illustrated in Figure 4, the laser serves as a high-energy beam source. Through photon-atom interaction, light energy is converted into internal energy within the material surface, intensifying atomic thermal vibration and establishing a local temperature gradient. This thermal effect alters the physical state of the material [41]. For hard and brittle materials, micro-zone softening or phase transformation is induced by thermal expansion, resulting in reduced brittleness [42]. For metallic materials, recovery and recrystallization may occur in the surface layer, leading to decreased hardness [43,44]. Subsequently, the high-pressure waterjet acts synchronously on the laser-pretreated area, leveraging the laser-induced “softening” to achieve efficient and controllable material removal.

Traditional waterjet machining relies on pure mechanical impact, where the removal mechanism for hard and brittle materials is dominated by brittle fracture, often causing edge chipping and micro-cracks. Conversely, while pure laser machining removes material via thermal evaporation, a recast layer or micro-cracks are easily formed in the heat-affected zone, and efficiency is limited by the laser scanning speed. These limitations are overcome by laser-mechanical synergy. The laser acts solely on the surface micro-zone. Laser energy and interaction time are precisely controlled to bring the surface layer to a critical processing state. At this point, the softened layer is rapidly cleared by the high-pressure impact of the waterjet, while damage to the underlying material caused by overheating is avoided.

#### 2.2.2. Acoustic-Mechanical Synergistic Reinforcement

**Figure 5:**
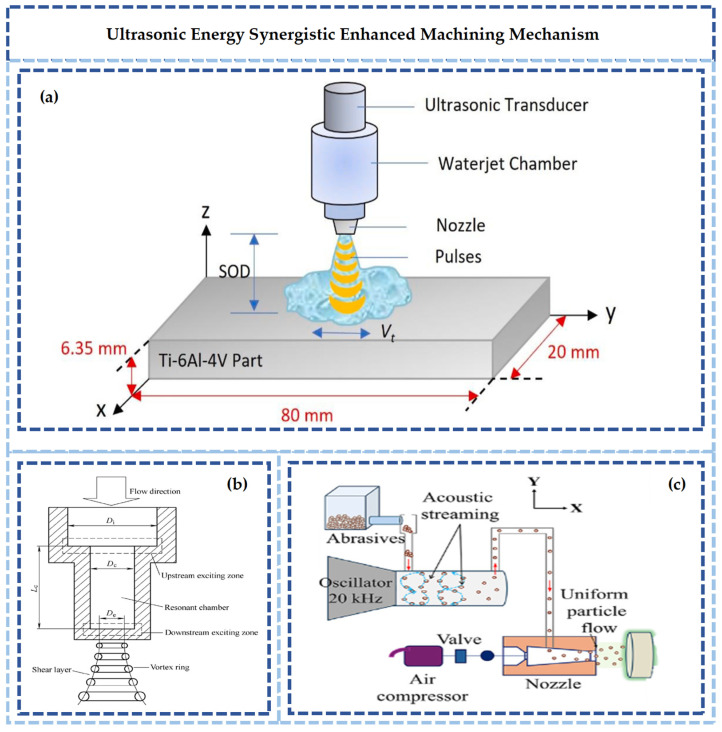
Schematic diagram of acoustic–mechanical energy synergistic strengthening: (**a**) Ultrasonic–Water Jet Machining System [45]. (**b**) Ultrasonic Resonant Cavity Schematic [46]. (**c**) Ultrasonic Abrasive Flow Control Mechanism [47].

The synergy between acoustic and mechanical energy is illustrated in Figure 5. An ultrasonic transducer transmits high-frequency mechanical vibrations to the waterjet nozzle, inducing periodic contraction and expansion within the fluid, inducing periodic contraction and expansion in the fluid within the nozzle [45]. Localized high-pressure and high-velocity micro-jets are released [46]. This cavitation jet not only enhances the impact capability of the waterjet but also achieves active regulation of the abrasive motion trajectory by matching the vibration frequency with the natural frequency of the material [47].

In traditional waterjet machining, jet morphology is limited by nozzle structure and pressure, presenting an uneven energy distribution of “dense center and divergent edges” [48], which leads to large fluctuations in surface roughness [49,50]. During deep cavity machining, jet energy decays exponentially with depth [51], making effective removal difficult to maintain [52,53]. These issues are optimized by acoustic synergy through the following mechanisms. First, the beam spot diameter is contracted by the periodic impact of ultrasonic vibration, enhancing the uniformity of energy density distribution and significantly improving surface roughness consistency. Second, the ultrasonic vibration accompanying the jet clears abrasive deposits on the inner wall of the nozzle, preventing clogging and extending continuous machining time. Third, resonance matching is achieved between the vibration frequency and the fracture toughness of the material [54].

#### 2.2.3. Magnetic-Mechanical Synergistic Reinforcement

**Figure 6:**
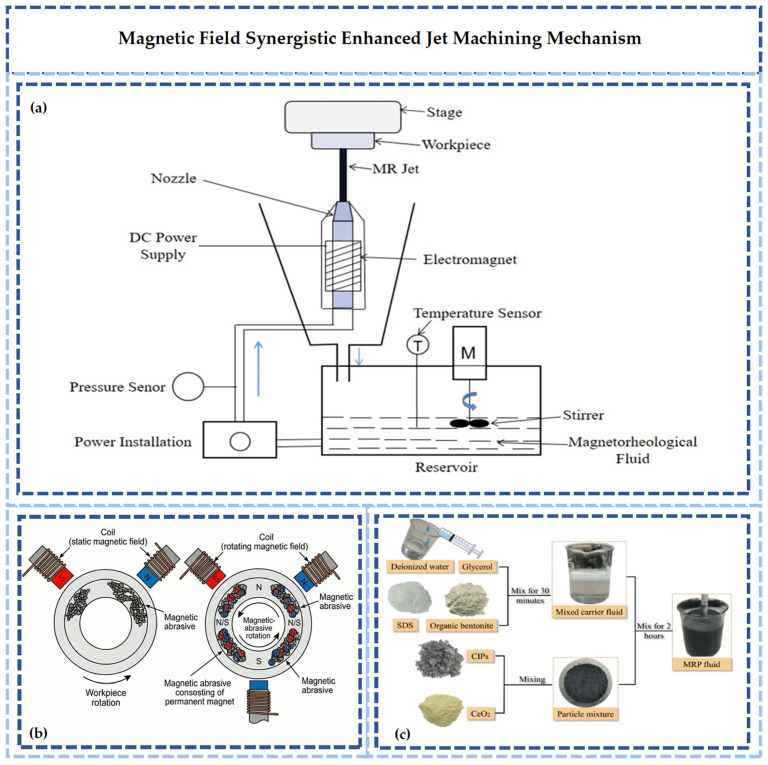
Schematic diagram of magnetic field–mechanical energy synergistic strengthening: (**a**) synergistic mechanism [24]. (**b**) schematic diagram of magnetic field contro. (**c**) preparation of magnetic fluid [55].

The synergistic mechanism of magnetic field energy and mechanical energy is shown in Figure 6. In traditional waterjet machining, abrasive distribution is affected by fluid turbulence. Random particle motion leads to inconsistent scratch depths on the machined surface, and subsurface damage layers can reach thicknesses of several micrometers. In contrast, for magnetic abrasives, a magnetorheological effect is induced by gradient magnetic fields generated via permanent magnets or electromagnetic coils [24]. Magnetic abrasives are aligned directionally under the magnetic field, forming a “high-density abrasive beam” along the gradient direction. Simultaneously, the trajectory of abrasive particles is altered by the Lorentz force, causing them to impact the target surface more concentratedly and reducing energy dissipation.

However, energy field-assisted waterjet machining is not a simple superposition of multiple energy fields. Instead, it aims at the synergy of material, structure, process, and performance. Adaptability between energy fields, tools, processes, and micro-structures must be considered. Input energy is regulated to overcome energy barriers, realizing the integrated manufacturing of micro-structure geometry and performance. The difficulty in theoretical and process innovation remains focused on the research of geometric precision of micro-structures and the evolution laws of the surface machining space under energy field coupling. The principles and typical applications of these typical energy field-assisted micro-grinding processes will be reviewed in the following sections.

## 3. Laser-Assisted Jet Machining Technology

### 3.1. Process Principle

Laser-assisted machining is characterized as a technique where high-efficiency, precision material removal is achieved through synergistic energy fields. The core of this technology is defined by the combination of the high energy density of lasers and the dynamic properties of fluid media. The laser is utilized as the primary energy source. High-precision energy input is provided. Photothermal or photochemical effects are induced on the material surface by the focused beam. Consequently, local melting, vaporization, or phase transformation is generated [56].

Simultaneously, a high-pressure fluid is introduced as an auxiliary medium. The softened material layer is stripped away by the fluid impact force [57]. Heat is rapidly removed via convective heat transfer. Thermal accumulation is suppressed. Surface quality is improved, material removal capability is enhanced, and thermal damage is reduced. Limitations of single-laser processing (excessive heat-affected zones) or pure fluid processing (insufficient efficiency) are overcome. Significant advantages are demonstrated in the micro-machining of hard and brittle materials, semiconductor wafer dicing, and precision surface modification. Characteristics such as high resolution [58], low damage, and strong controllability are exhibited [59].

**Figure 7:**
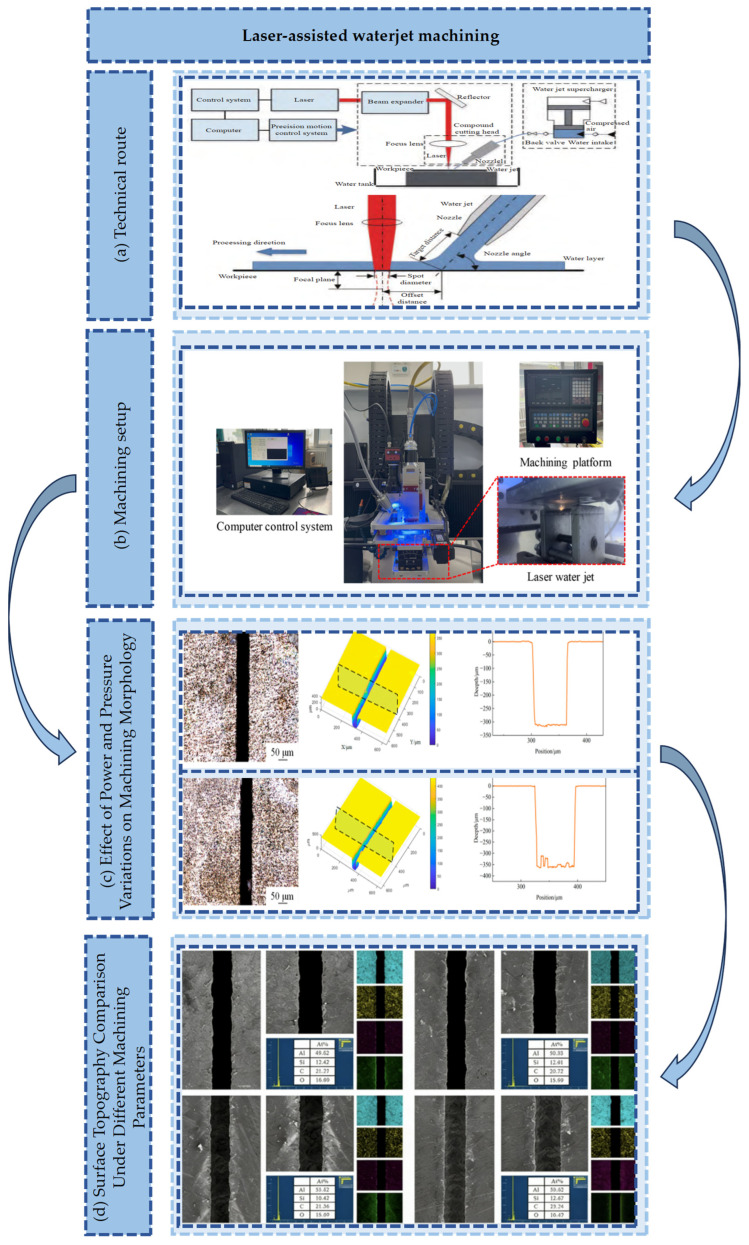
Laser−assisted waterjet machining: (**a**) Technical route [64]. (**b**) Machining setup [57]. (**c**) Effect of Power and Pressure Variations on Machining Morphology [44]. (**d**) Surface Topography Comparison Under Different Machining Parameters [44].

The laser-assisted waterjet machining technology is illustrated in Figure 7a. The machining apparatus is shown in Figure 7b. Processing morphologies under different water pressures and energy levels are presented in Figure 7c. Ablation morphologies under varying parameters are depicted in Figure 7d. As a key research direction in advanced manufacturing, the “thermal-mechanical” coupling is investigated in basic theoretical studies. Three-dimensional transient temperature field models and material ablation kinetics models are established. The coupling laws between laser energy absorption, material thermal softening, and waterjet impact shear are revealed. Specifically, the local material temperature is raised to the critical softening point by nanosecond or picosecond laser pulses. Yield strength is significantly reduced. Subsequently, plastic removal of the softened material is achieved by the high-pressure waterjet via hydrodynamic pressure and shear effects. Thermal damage caused by material melting and vaporization is effectively avoided. A theoretical basis for the low-damage processing of hard, brittle, and heat-sensitive materials is provided.

Regarding process optimization [60], the interaction between laser and waterjet parameters is quantified via Response Surface Methodology (RSM) and Finite Element Method (FEM) [61]. For instance, in the processing of semiconductor materials (e.g., single-crystal silicon, 4H-SiC), micro-grooves with aspect ratios exceeding 5:1 are obtained when specific ratios of laser power to waterjet pressure are reached [62]. The thickness of the Heat Affected Zone (HAZ) is controlled at the micron level [63]. For ceramic matrix composites, simultaneous removal of fibers and the matrix is achieved by regulating the laser scanning path and waterjet impact timing [64]. Interface delamination and fiber pull-out are suppressed [65]. A process window optimization method based on the “thermal-mechanical” synergistic mechanism is established. Material removal rates are increased by over 30% [66], compared to traditional laser machining. Surface roughness is reduced to the sub-micron level [67,68].

Table 1 summary and analysis relevant studies on laser-assisted jet machining covering process parameters and key research conclusions [69]. Current research is primarily focused on thermal damage control in hard and brittle materials, such as gallium arsenide, silicon carbide, and gallium oxide. The core advantages of laser-assisted waterjet technology are verified from various perspectives. Machining quality is significantly improved through the synergy of localized laser thermal softening and the immediate cooling and removal functions of the waterjet. The unique value of this technology in achieving micron-level topography control and special functional surface preparation is revealed.

### 3.2. Typical Applications

In the field of semiconductor manufacturing, the technology is successfully applied to hard and brittle materials such as single-crystal silicon and silicon carbide. It is indicated by research results that near-zero thermal damage micro-grooves and cutting are achieved on semiconductor wafers via laser-waterjet synergy. Recast layers and micro-cracks are eliminated from the surface. The electrical integrity of the material is effectively maintained.

In the aspect of advanced ceramic processing, limitations of traditional methods are overcome for engineering ceramics such as silicon nitride and zirconia [74]. High-quality machining of high-aspect-ratio micro-channels is realized. A dense surface structure is obtained. Typical thermal cracking issues in ceramic materials are avoided. A new pathway for the precision manufacturing of ceramic components is provided.

In the field of composite material treatment, unique advantages are demonstrated, particularly in the processing of carbon fiber reinforced polymers and ceramic matrix composites. Fiber pull-out, delamination, and interface damage are effectively solved through precise control of energy input and waterjet impact parameters. High-quality machining of holes and contours is achieved.

In the aspect of biomedical device manufacturing, the technology is utilized for micro-machining biocompatible materials such as zirconium-based amorphous alloys. It is shown by research results that functional surfaces with specific micro-textures are fabricated via a low-temperature softening removal mechanism. Material crystallization is avoided. New surface functionalization schemes for medical equipment like surgical instruments are provided [75].

In summary, comprehensive improvement in machining quality—from macroscopic morphology control to microscopic structure preservation—is achieved by these studies through multi-field coupling effects of laser and waterjet. The transition of this technology from laboratory research to industrial application is promoted.

## 4. Ultrasonic-Assisted Jet Machining Technology

### 4.1. Process Principle

High-frequency ultrasonic vibration is integrated with high-pressure waterjet technology in ultrasonic-assisted waterjet machining. Ultrasonic energy is directly injected into the nozzle. Consequently, a periodic pulse effect is generated at the exit of the continuous waterjet, leading to dynamic pressure fluctuations [76]. The impact of abrasive particles is significantly enhanced by the coupling of ultrasonic vibration and jet kinetic energy. Simultaneously, cavitation effects are induced. Micro-jet impacts are generated, resulting in a synergistic energy mechanism. Uniform abrasive distribution, enhanced jet focusing, and efficient material removal are achieved by regulating ultrasonic parameters such as frequency and amplitude, alongside jet parameters including pressure and standoff distance. The method is particularly utilized for the precision machining of hard and brittle materials, such as ceramics and glass. Thermal damage is effectively mitigated while efficiency is improved. A solution is thus provided for the ultra-precision manufacturing of complex surfaces and micro-structures.

Relevant research on ultrasonic-assisted waterjet machining is summarized in Table 2. It is indicated that the velocity of micro-jets generated during bubble collapse is significantly enhanced by ultrasonic waves within specific frequency ranges. A distinct secondary acceleration of abrasive particles within the ultrasonic vibration field was observed via Particle Image Velocimetry technology. The material removal process is significantly influenced by this dynamic behavior.

**Figure 8:**
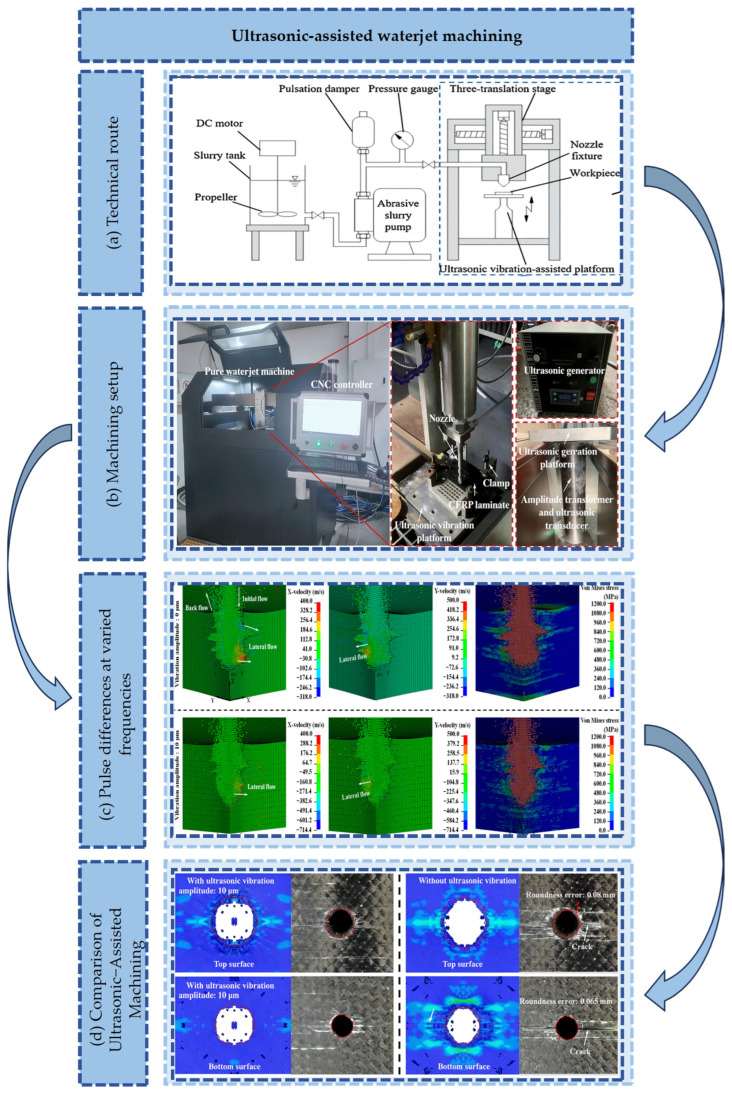
Ultrasonic−assisted waterjet machining: (**a**) Technical route [22]. (**b**) Machining setup [76]. (**c**) Pulse differences at varied frequencies [76]. (**d**) Comparison of Ultrasonic-Assisted Machining [76].

Ultrasonic-assisted waterjet machining technology is illustrated in Figure 8a. The ultrasonic-assisted jet machining device is presented in Figure 8b. Pulse differences at varied frequencies are displayed in Figure 8c. Figure 8d illustrates the machining comparison between conventional waterjet and ultrasonic-assisted waterjet. The broad application prospects of ultrasonic-assisted machining technology in precision manufacturing are demonstrated by these studies [87,88]. Current research is primarily focused on the modulation of jet dynamic characteristics by ultrasonic vibration [45]. Ultrasonic energy is converted into high-frequency pressure pulsations. Consequently, abrasive particle agglomeration is effectively disrupted, and jet turbulence kinetic energy is enhanced. The water cushion effect prevalent in traditional abrasive waterjet machining is significantly mitigated by this modulation. This modulation significantly improves jet energy utilization. Regarding material removal mechanisms, ultrasonic vibration promotes the transition of hard and brittle materials from brittle fracture to ductile removal [89,90]. This process is closely correlated with the matching degree between the jet pulsation frequency and the natural frequency of the material [91,92].

In terms of process parameters, complex interactions between vibration parameters and jet parameters—including impact angle and nozzle movement direction—are revealed through systematic optimization [93]. It is found that material removal efficiency is enhanced by increasing amplitude within a specific range. However, jet morphology is altered by excessive amplitude, negatively affecting machining accuracy [76]. The regulation mechanism of ultrasonic vibration on flow field characteristics is further elucidated by numerical simulations based on computational fluid dynamics. The significant influence on pressure distribution in the stagnation zone is particularly highlighted. Unique advantages are demonstrated by ultrasonic-assisted technology in the surface strengthening of metallic materials. A beneficial stress distribution state is induced in the material surface layer, while surface properties are improved. It is indicated by research on biomedical materials that biocompatibility is effectively enhanced. Significant application potential is also exhibited in metal anti-corrosion treatments. Furthermore, regarding quality monitoring, the amplitude and frequency spectrum of vibration signals are closely correlated with cutting quality and penetration rate. Surface roughness during the machining process is effectively predicted and controlled via optimized wavelet parameters and feature extraction methods. It is noted that wavelet packet transform is superior to traditional time-domain analysis methods in predicting maximum roughness.

### 4.2. Typical Applications

Ultrasonic-assisted waterjet machining, as an important precision manufacturing innovation, has witnessed significant advances in both theory and application. In aerospace manufacturing, this technology is adopted for the precision repair of turbine blade thermal barrier coatings. High-frequency, low-amplitude vibration regulates the jet shearing effect, enabling high-precision coating removal without damage to the superalloy substrate, while effectively controlling the surface roughness of complex curved structures. In the biomedical sector, it is applied to the surface functionalization of orthopedic implants. Cross-trajectory jet impact is employed to construct multi-level microporous structures on titanium alloy surfaces, which significantly boosts osteoblast activity and improves implant biocompatibility. In micro-nano optical manufacturing, high-quality machining of glass-based microfluidic chip channels is implemented. Ultrasonic vibration optimizes the channel cross-section geometry, enhances sidewall verticality and ensures bottom surface quality, offering a novel process route for the precision fabrication of microfluidic chips.

The unique advantages of ultrasonic-assisted abrasive waterjet machining technology in resolving precision machining challenges are fully demonstrated by these research findings. Irreplaceable value is currently exhibited in high-end manufacturing fields—specifically for biomedical implants, optical glass components, semiconductor substrates, and aerospace composite materials—where stress-free damage, high surface integrity, and complex microstructure machining are required. Broader applications in strategic emerging industries, such as precision medical devices, new energy equipment, and integrated circuits, are anticipated with the optimization of intelligent control algorithms and the development of novel hybrid machining processes. The technology is poised to become a key support for the transformation and upgrading of high-end equipment manufacturing.

## 5. Magnetic Field-Assisted Jet Machining Technology

### 5.1. Process Principle

In magnetic field-assisted abrasive waterjet machining, an external magnetic field precisely controls the trajectory of ferromagnetic abrasives [55,94]. A quadrupole alternating magnetic field is applied at the nozzle periphery. A magnetic lens effect is formed as magnetic field lines penetrate the jet. Abrasives are driven toward the axis by magnetic driving forces, causing the jet diameter to shrink [95]. Simultaneously, abrasives are induced to align head-to-tail, forming a directional chain-like structure [96]. The concentration of impact angles is thereby improved. Subsurface micro-cracks and shear spalling are induced by stress wave penetration. Through this synergistic action, the cold precision machining of hard and brittle materials is achieved under the flushing of the cooling medium.

Relevant research on magnetic field-assisted jet machining is summarized in Table 3. Through systematic experiments and theoretical modeling, that the motion behavior and machining performance of the abrasive jet are effectively regulated by the magnetic field. Long-distance stable transmission of the jet was achieved in early studies via axial magnetic field control. It was discovered that the focusing effect is determined by the product of the magnetic gradient force and particle magnetization intensity. Machining accuracy was promoted to the sub-nanometer level in subsequent work by optimizing the magnetic field distribution function. The technology was successfully applied to the machining of complex shapes, such as aspheric optical components. The motion characteristics of abrasive particles under the magnetic field were quantitatively analyzed using Particle Image Velocimetry and high-speed photography. Magnetohydrodynamic models were established. A non-linear relationship between magnetic field intensity and material removal rate was revealed. A theoretical basis for process parameter optimization is thus provided [97].

**Figure 9:**
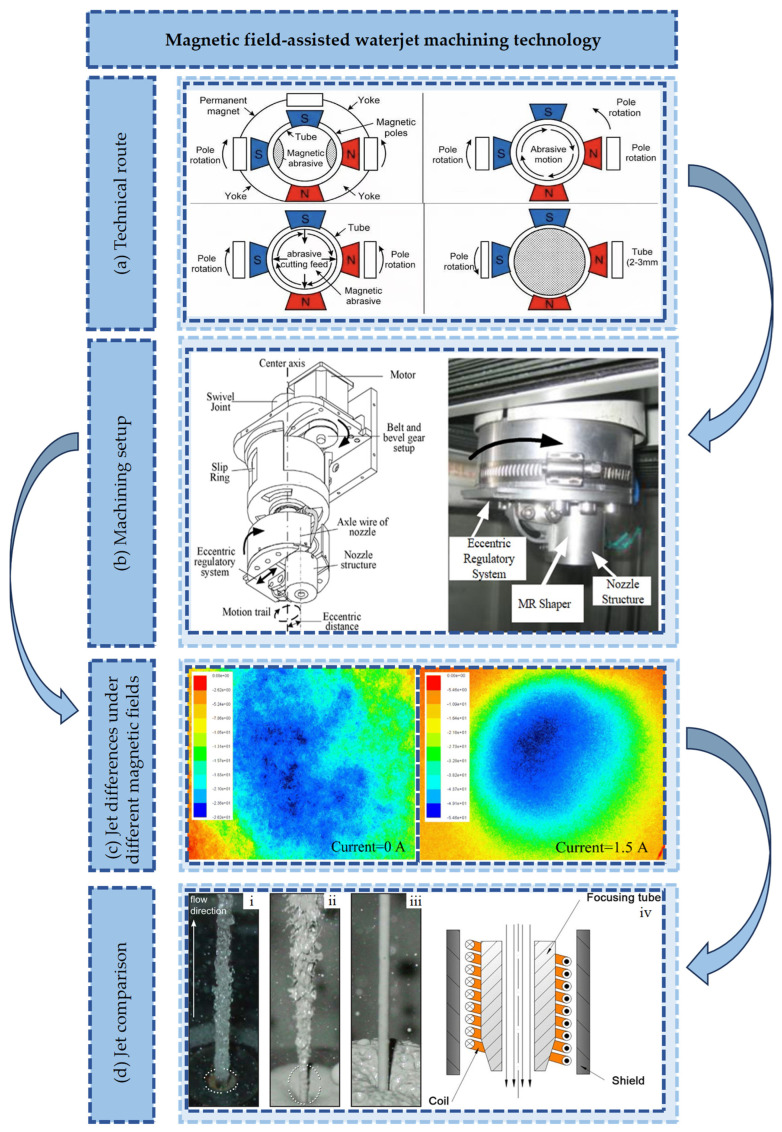
Magnetic field-assisted waterjet machining technology: (**a**) Technical route. (**b**) Machining setup [24]. (**c**) Jet differences under different magnetic fields [95]. (**d**) Jet comparison [95].

Research on magnetic field-assisted jet machining centers on the precise control of abrasive trajectory and energy distribution via magnetic fields. Jet divergence is effectively inhibited. Machining efficiency and surface quality are enhanced. The physical limitations of traditional jet machining are transcended, opening new avenues for high-precision manufacturing. Magnetic field-assisted machining technology is illustrated in Figure 9a. The magnetic field-assisted jet machining device is presented in Figure 9b. Jet differences under varied magnetic fields are displayed in Figure 9c. A feature comparison between conventional jets and magnetic field-assisted jets is shown in Figure 9d. The integration of ultrasonic vibration with magnetic field assistance has been investigated. A reduction in contact stiffness between abrasives and the workpiece is induced by the compound field via cavitation effects. Material removal efficiency is notably increased; this is particularly advantageous for hard material machining. Regarding abrasive formulations, the influence of magnetic particle volume fraction and particle size distribution on machining performance has been investigated. The standardization of magnetic polishing fluids is promoted [104,105]. Synergistic optimization of magnetic responsiveness and abrasive hardness is realized through the design of core-shell structured abrasives and the regulation of shell thickness. Furthermore, abrasive trajectories under multi-pole magnetic fields are predicted using finite element simulation. Theoretical guidance for the machining of complex curved surfaces is provided [106].

### 5.2. Typical Applications

In aerospace manufacturing, stringent surface quality standards are imposed by precision components with complex curved surfaces, such as turbine blades. The limitations of traditional machining—poor adaptability and surface damage—are addressed by abrasive jet technology based on magnetic field regulation. A stable, focused beam is generated from the abrasive jet containing magnetic particles via axial magnetic field application. Jet divergence during long-distance transmission is effectively curbed. Abrasive particle trajectories are precisely guided. Machining efficiency is concurrently elevated through the enhancement of internal jet forces.

The adaptability of this technology to complex blade geometries is confirmed by application results. Uniform material removal is attained even in difficult-to-machine areas, such as blade tips and tenon grooves. High surface quality is secured [107]. Batch production has been successfully implemented, with marked improvements in efficiency and yield. The potential of magnetic field-assisted jet technology in resolving precision machining challenges for complex curved surfaces is substantiated. A reliable solution for the green, efficient manufacturing of high-value components is thus presented [103].

## 6. Conclusions and Outlook

### 6.1. Conclusions

Waterjet technology is utilized in complex surface machining due to advancements in ultra-precision technology and demand in sectors such as aerospace components, semiconductor packaging, and optical micro-structures. Advantages including the absence of thermal damage and broad material adaptability are offered. However, multi-energy field-assisted waterjet technology is currently at the stage of fundamental mechanism exploration. In the context of high-performance manufacturing, technical challenges in waterjet machining regarding machinability, micro-scale accuracy, structure adaptation, and the surface integrity-efficiency trade-off are discussed. As shown in Table 4, this paper conducts a comprehensive comparison of the technical parameters, fundamental performance and machining performance of various energy field-assisted jet technologies. Coupling mechanisms between multi-energy fields and waterjets are analyzed. The development status of laser-, ultrasonic-, and magnetic field-assisted technologies is reviewed, with a focus on process principles and typical applications. The following conclusions are drawn:

Technical challenges in high-performance manufacturing using traditional waterjets are analyzed: Research indicates that brittle fracture and micro-crack damage are induced by a single mechanical energy field when machining hard, brittle materials. Profile distortion and accuracy loss in high-aspect-ratio structures are caused by jet diffusion and energy attenuation. Furthermore, a constraint exists between surface integrity and machining efficiency.

Synergistic enhancement mechanisms of mechanical-special energy fields are elucidated: Local thermal softening is achieved by laser assistance via “photothermal pre-treatment,” promoting the brittle-to-ductile removal transition. Abrasive kinetic energy is enhanced and flow field stability within narrow spaces is improved by ultrasonic assistance via cavitation and high-frequency pulses. Active beam focusing and directional removal are realized by magnetic field assistance, where magnetic abrasives are constrained by magnetic driving forces.

Process advantages and limitations of typical assisted technologies are evaluated: While laser, ultrasonic, and magnetic fields serve as auxiliary means to enhance waterjet performance, significant limitations exist:

The core challenge of laser-assisted waterjet technology lies in energy coupling efficiency and process control complexity. Severe energy attenuation results from strong laser absorption and scattering by the water medium, particularly in the infrared band. Consequently, effective machining depth is limited, and beam quality is degraded. Simultaneously, difficult-to-control recast layers are formed on sidewalls by molten material generated during jet impact.

The primary drawbacks of ultrasonic-assisted technology involve a narrow parameter window and structural impacts. Although jet impact force and material removal rates are enhanced by ultrasonic-induced cavitation, excessive vibration is introduced by high amplitudes. This conversely leads to increased roughness and decreased geometric accuracy.

Distinct limitations are exhibited by magnetic field-assisted technology. Effectiveness is highly dependent on workpiece electromagnetic properties. Optimization is currently demonstrated only in micro-hole machining of specific composites Advantages are mostly established under low pulse energy; universality across broader material systems and high-energy conditions is lacking. Furthermore, nozzle system complexity is inevitably increased by integrating ultrasonic components, raising manufacturing costs and potentially affecting reliability. These inherent defects constrain large-scale application in precision scenarios.

### 6.2. Outlook

Despite significant progress in multi-energy field-assisted technology, deep exploration in the following dimensions is required for industrial adoption:

(1) Mechanisms of Multi-Physics Non-linear Coupling and Cross-Scale Modeling. Future research prioritizes the non-linear synergistic mechanisms between heterogeneous energy fields. Cross-scale modeling frameworks bridging Molecular Dynamics (MD) and Computational Fluid Dynamics (CFD) capture atomic-level material removal behaviors within complex thermo-mechanical-acoustic-magnetic environments. A key focus lies in high-fidelity simulations of cavitation bubble collapse dynamics under magnetic constraints and the resulting micro-jet impact intensity. Furthermore, the energy matching windows for sequential coupling—such as “nanosecond laser thermal softening followed by magnetorheological jet polishing”—are quantitatively defined to optimize energy efficiency and surface integrity.
(2) AI-Driven Process Architectures and Digital Twin Systems. To address the stochastic nature of jet-material interactions, research shifts towards data-driven process intelligence. This involves integrating Deep Reinforcement Learning (DRL) with multi-modal sensing—such as Acoustic Emission (AE) and high-speed shadowgraphy—to establish real-time feedback loops. Digital Twin models enable the prediction of material damage response and the closed-loop modulation of laser power, ultrasonic frequency, and jet pressure. This approach is instrumental for achieving consistent machining quality in heterogeneous materials like Ceramic Matrix Composites (CMCs) and Gallium Nitride (GaN).
(3) Integration of High-Reliability Equipment with In-Situ Metrology. The engineering focus involves developing compact, coaxial integrated nozzle systems for stable operation under extreme conditions. Technical developments address the water-resistant coupling of high-power laser paths and the fatigue mitigation of ultrasonic transducers under high-pressure water-hammer effects. Next-generation equipment embeds in-situ optical sensors (e.g., white-light interferometry or confocal microscopy) to allow for “machining-while-measuring.” This integration minimizes repositioning errors and maintains the structural integrity of core components throughout extended high-frequency service.
(4) Expansion into “Shape-Performance Integrated” Manufacturing for Extreme Applications. The research paradigm shifts from basic geometric shaping toward “Geometry-Property Synergistic” manufacturing. In the semiconductor sector, applications include sub-nanometer thinning and dicing of 4H-SiC and diamond substrates to achieve zero subsurface damage (SSD) and atomic-scale flatness. Within aerospace engineering, the technology enables the fabrication of complex cooling holes and bionic drag-reduction textures on turbine blades, while facilitating the tailoring of surface residual stress fields to improve fatigue life. This technology is positioned as a strategic platform for the high-integrity manufacturing of critical components operating under extreme thermal and mechanical loads.

## Figures and Tables

**Table 1:** Relevant researches on laser-assisted waterjet machining technology.

Materials	Research Topics	Hybrid Processes	Main Findings	Key Open Issues	References
**Silicon Nitride**	Laser-assisted waterjet micro-milling	Laser-waterjet	Enables nearly damage-free micro-milling by laser softening and waterjet cooling.	Inconsistent groove depth due to waterjet turbulence; unclear removal mechanism.	[61]
**Zr-based Amorphous Alloy**	Non-crystallization micromachining	Laser-waterjet	Achieves crystallization-free micromachining; waterjet prevents oxidation and crystallization.	Risk of local crystallization at high energy; trade-off between efficiency and quality.	[70]
**SiC**	Coaxial gas-assisted laser waterjet machining	Laser-waterjet-Gas	Gas stabilizes the waterjet, enabling high-aspect-ratio, damage-free cutting.	Low efficiency; incomplete water removal in deep grooves; interfacial effects unknown.	[71]
**Silicon**	Laser waterjet dicing	Laser-waterjet	Reduces thermal damage; waterjet efficiently removes molten material.	Mechanism of melt ejection; control of laser self-focusing.	[18]
**Silicon Wafer**	Synergistic sidewall post-processing	Laser-Abrasive	Combined method reduces sidewall roughness via laser-enhanced conductivity and cavitation.	Optimization of multi-parameter synergy for higher efficiency and precision.	[72]
**Single-crystal Silicon**	Hybrid laser-waterj et ablation	Laser-waterjet	Enables material removal below melting point, minimizing thermal damage.	Unclear laser-waterjet coupling mechanism; need for broader material application.	[67]
**Al alloy 2024-T3**	Laser-induced plasma electrolyte jet machining	Laser Plasma-Electrolyte Jet	Synergy of laser and electrolyte jet significantly increases surface hardness via grain refinement.	Unclear plasma-jet coupling mechanism; need to extend to other metals.	[73]
**Ti-6Al-4V**	Laser ablation under flowing water layer	waterjet-Underwater Laser	Produces narrow, deep grooves with minimal heat-affected zone under a thin water layer.	Need to optimize water flow to avoid laser blocking; extend to other materials.	[59]

**Table 2:** Related studies of ultrasonic-assisted waterjet machining.

Materials	Research Topics	Hybrid Processes	Main Findings	Key Open Issues	References
**AA6060**	Acoustic Chamber Length on Erosion	Ultrasonic Pulsed waterjet	A hyperbolic relationship was observed between erosion depth, chamber length and standoff distance.	Synergistic parameter selection is needed for specific processing objectives.	[77]
**AA7075-T6**	Surface Integrity	Ultrasonic Impact-Special Pulsed waterjet	Surface roughness, microhardness and residual compressive stress were improved; single process failed to optimize all properties.	Synergistic multi-technology surface modification requires development.	[78]
**AISI 304**	Surface Treatment Hardening	Ultrasonically Generated Pulsed waterjet	Tensile residual stress was converted to compressive, and treated zone hardness was increased.	Potential as a novel surface treatment was demonstrated.	[79]
**Quartz Glass**	Material Removal Surface Quality	Ultrasonic Vibration-Assisted waterjet	Ultrasonic vibration improved material removal rate and erosion depth; pressure and amplitude were key factors.	Mechanism of sub-surface damage suppression under high-frequency impact.	[80]
**AISI 304 Welded Joint**	Stress, Hardness, Roughness & Microstructure	Ultrasonic Pulsed waterjet Peening	Residual stress and roughness increased; subsurface hardness improved, and plastic deformation was confirmed.	Suitable for welded structures needing improved fatigue and corrosion resistance.	[81]
**AA7075**	Residual Stress Prediction Microstructure	Ultrasonic Impact-waterjet	A validated residual stress prediction model was established.	Model generalization for complex geometries beyond standard planar coupons.	[82]
**Pure Al, Al-Cu Alloy**	Stress, Strength Corrosion Resistance	SFN-MFC	Central erosion of pure Al was suppressed; roughness reduced, and oxide film formed on Al-Cu alloy.	Quantitative correlation between micro-texture parameters and long-term osteoblast adhesion.	[83]
**Ti** **-** **6Al** **-** **4V**	Surface Morphology for Osseointegration	Ultrasonic Pulsed waterjet (20/40 kHz)	Surface topography was superior to traditional techniques for bio-interaction.	Suitable as a low-temperature surface treatment for implant performance improvement.	[84]
**Glass**	Microchannel Machining	Ultrasonic Vibration-Assisted Abrasive waterjet	Material removal rate and channel size improved; wall inclination reduced, bottom quality unchanged.	Suitable for efficient micro-machining with guaranteed surface quality.	[85]
**K9 Glass**	Micro-Hole Machining Morphology	Ultrasonic Vibration-Assisted Abrasive waterjet	Material removal rate and bottom morphology improved; W-shaped bottom was eliminated.	Mechanism applicable for improving ultrasonic abrasive waterjet polishing uniformity.	[86]

**Table 3:** Related studies on magnetic field-assisted jet machining technology.

Materials	Research Topics	Hybrid Processes	Main Findings	Key Open Issues	References
**Flexible Magnetic Abrasive (Elastomer-containing)**	Flexible Magnetic Abrasive Jet Machining Properties	Magnetic Field-Assisted Jet	Magnetic field constrained jet direction, improved machining uniformity and surface roughness through sliding friction.	Complex geometry machining performance requires verification.	[98]
**Magnetorheological Polishing Fluid, K9 Glass**	Eccentric Rotational Vertical Jet Removal	Magnetic Field-Assisted Jet	Ideal Gaussian-like removal function was achieved at 0.8× characteristic length eccentricity.	Eccentric model applicability and path planning for complex surfaces need research.	[99]
**Magnetorheological Microjet Fluid, 5052 Al Alloy**	Material Removal: Simulation Process Optimization	Magnetic Field-Assisted Jet	A reliable CFD model was optimized to reduce Ra from 355 nm to 253 nm.	Model generalization for diverse materials/fluids needs further study.	[100]
**Magnetorheological Jet Polishing Fluid**	Complex Optical Element Deterministic Polishing	Magnetic Field-Assisted Jet	Nanoscale precision was achieved, with insensitivity to nozzle-workpiece distance.	Large/high-steepness free-form surface polishing efficiency needs improvement.	[101]
**Magnetic Abrasive (Magnetic/Abrasive Grains)**	Magnetic-Assisted Precision Machining Progress Review	Multi-Auxiliary Energy Combined Machining	Magnetic-assisted machining reaches hard-to-process areas but faces accuracy challenges.	Intelligent adaptive systems for complex parts are lacking.	[102]
**SPION Magnetic Nanoabrasive, BK-7 Glass**	Intelligent Recyclable Nanoabrasive Development	Nano Magnetic-Abrasive Integration	High-magnetization recyclable nanoabrasive reduced Ra from 411 nm to 22.3 nm.	Abrasive long-term recycling, magnetic loss and economy need study.	[103]

**Table 4:** Comprehensive comparison of technical specifications, capabilities, and machining performance of various energy field-assisted jet technologies.

Comparison Criteria	Conventional WJ/AWJ	Laser-Assisted Jet Machining (LAJM)	Ultrasonic-Assisted Jet Machining (UAJM)	Magnetic-Assisted Jet Machining (MAJM/MJP)
**Energy Field Mechanism**	Mechanical kinetic impact	Photothermal preheating + Mechanical erosion	Acoustic cavitation + Periodic pulsation	Magnetic confinement + Gradient focusing
**Key Physical Effects**	Brittle fracture/Micro-cutting	Thermal softening & shear strength reduction	Micro-jets from cavitation & enhanced particle momentum	Magnetorheological effect & trajectory orientation
**Material Removal Rate (MRR)**	• ≤2 mm^3^/s (General) [5] • 345.8 mm^3^/min (Ti-6Al-4V) [5] • 150 mm^3^/min (Ni-composite) [6]	• A significant increase occurs at critical resolved shear stress < 5 MPa (1650 K) [27]	• Up to +82% (Ceramics) [35] • ~+20% (CFRP drilling) [76]	• 53.051 mm^3^/min (K9 glass) [6] • 1.974 μm/h (Sapphire, UAMP) [94]
**Surface Roughness (Ra)**	• 1–5 μm [6] • 0.537 μm (Optimized, Ti6Al4V) [108]	• Greatly reduced (smoother surface finish) [27]	• 0.75 μm → 0.32 μm (CFRP hole) [86]	• 4.86 nm (K9 glass) [24] • ~0.442 nm (Sapphire) [94] • 355 → 253 nm (Sa, Al alloy) [100]
**Technical Capabilities & Advantages**	• No HAZ [5] • Thickness: ≤ 304.8 mm [5]	• Effective for difficult-to-cut hard-brittle materials	• Stable machining of deep holes and narrow grooves	• Minimal damage to non-machining zones • High focusing
**Technical Limitations**	Trade-off between efficiency and precision	Potential secondary thermal damage	Complex transducer integration; Impedance matching issues	Limited to magnetic abrasives; Constraint in magnetic field design
**Main Applications**	General metals, composites	Ceramics, quartz glass, high-strength alloys	Precision hole drilling, microfluidic chips	Ultra-precision polishing, high-aspect-ratio microstructures

## Data Availability

No data for public archival are reported in this study.
